# Supplementary material for: PEDF-Mediated Mitophagy Triggers the Visual Cycle by Enhancing Mitochondrial Functions in a H2O2-Injured Rat Model
Source: Cells. 2021 May 6;10(5):1117. doi: 10.3390/cells10051117 (PMC8148157; doi:10.3390/cells10051117)
Supplement: Supplementary file 1 [file cells-10-01117-s001.zip › cells-1185730-supplementary.pdf]

Figure S1

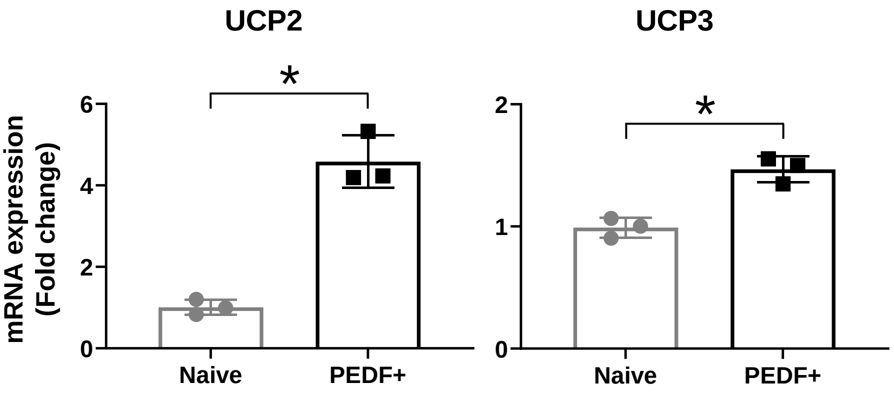

Figure S1. The mRNA expression of mitochondrial biogenesis markers (e.g., UCP2 and UCP3) in naïve PD-MSCs (Naïve) and PD-MSCs^{PEDF} (PEDF+) by qRT-PCR. The data from each group are shown as the mean ± SD and were analyzed by Student’s t-test. *$p<0.05$ vs. naïve PD-MSCs.

Figure S2

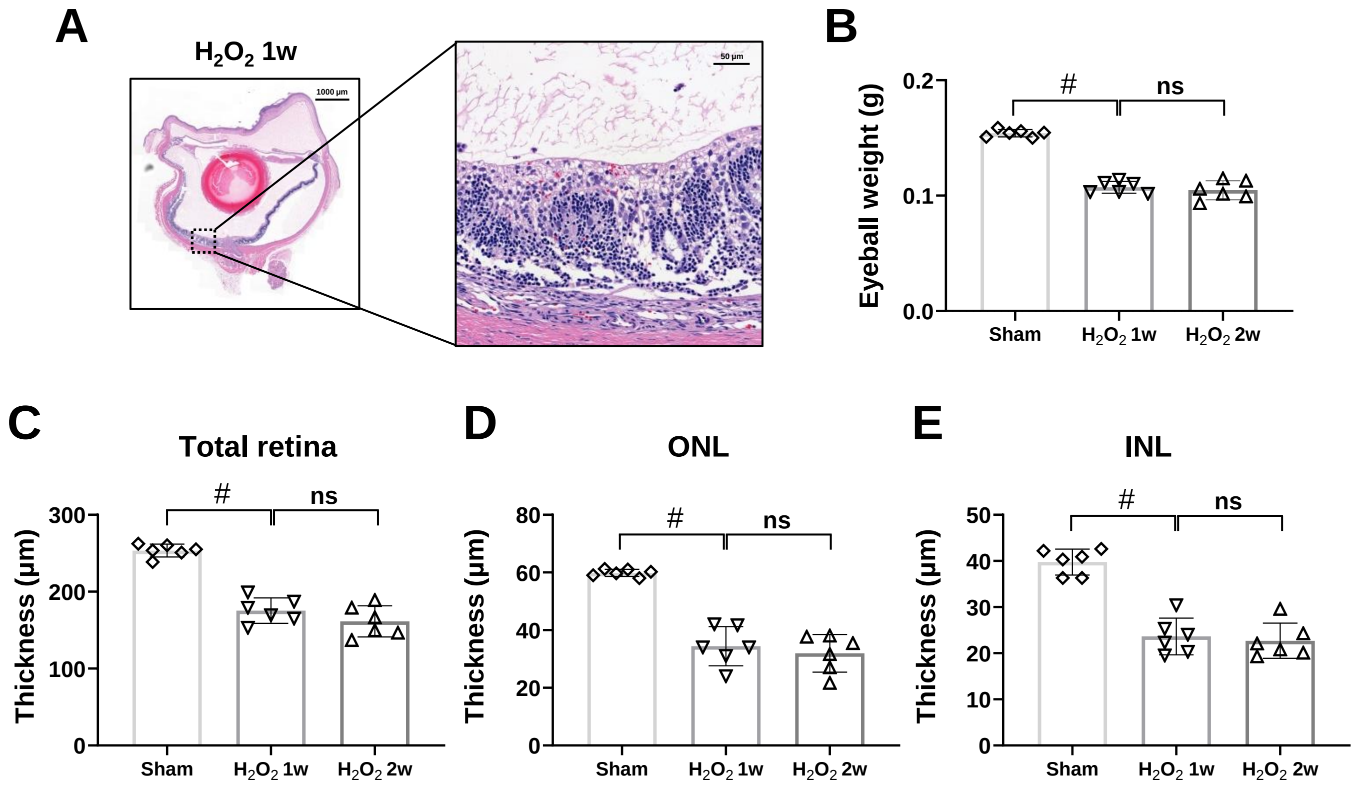

Figure S2. H_2O_2-injured rat modeling for 1 and 2 weeks. (**a**) Histological images in H&E-stained rat eyeball sections for 1 week. (**b**) Eyeball weight (gram; g) in H_2O_2-injured 1 and 2 weeks (n=6/group). Measurement of (**c**) the total retina, (**d**) outer nuclear layer (ONL), and (**e**) inner nuclear layer (INL) in H&E-stained sections (n=6/group). The data represent the mean ± SD and were analyzed by one-way ANOVA. #p<0.05 vs. Sham.

Figure S3

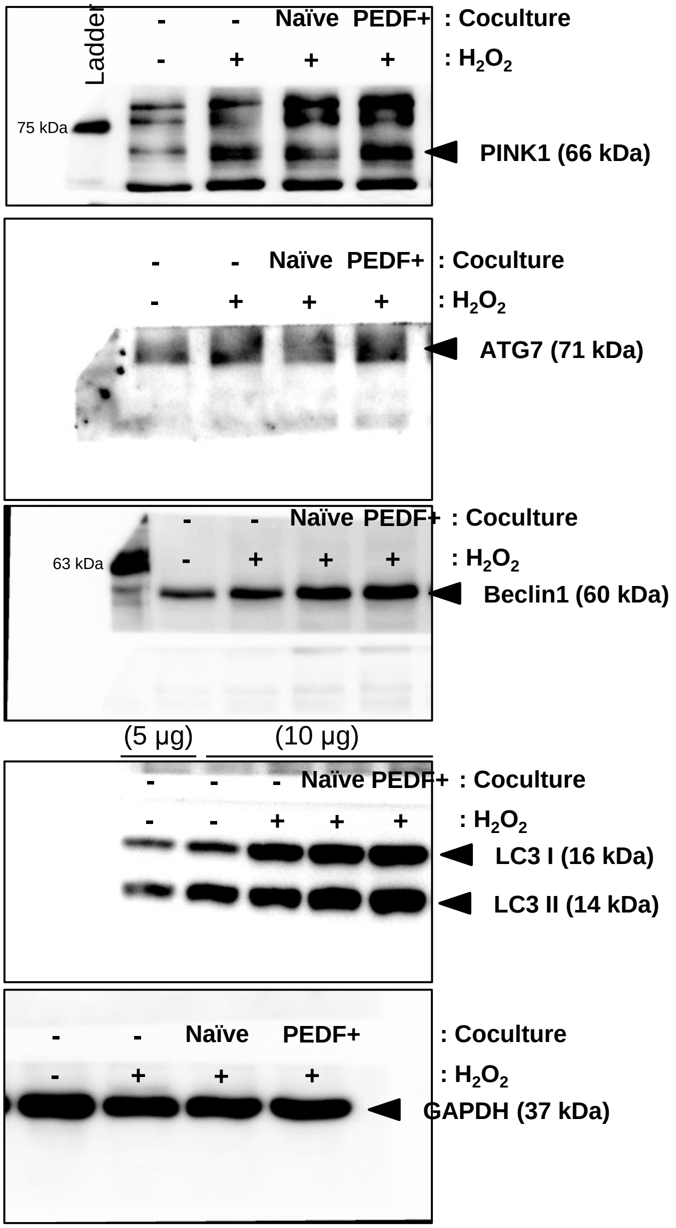

Figure S3. Uncropped membrane images by western blotting in H_2O_2-injured ARPE-19 cells cocultured with naïve PD-MSCs (Naïve) and PD-MSCs^{PEDF} (PEDF+)

Figure S4

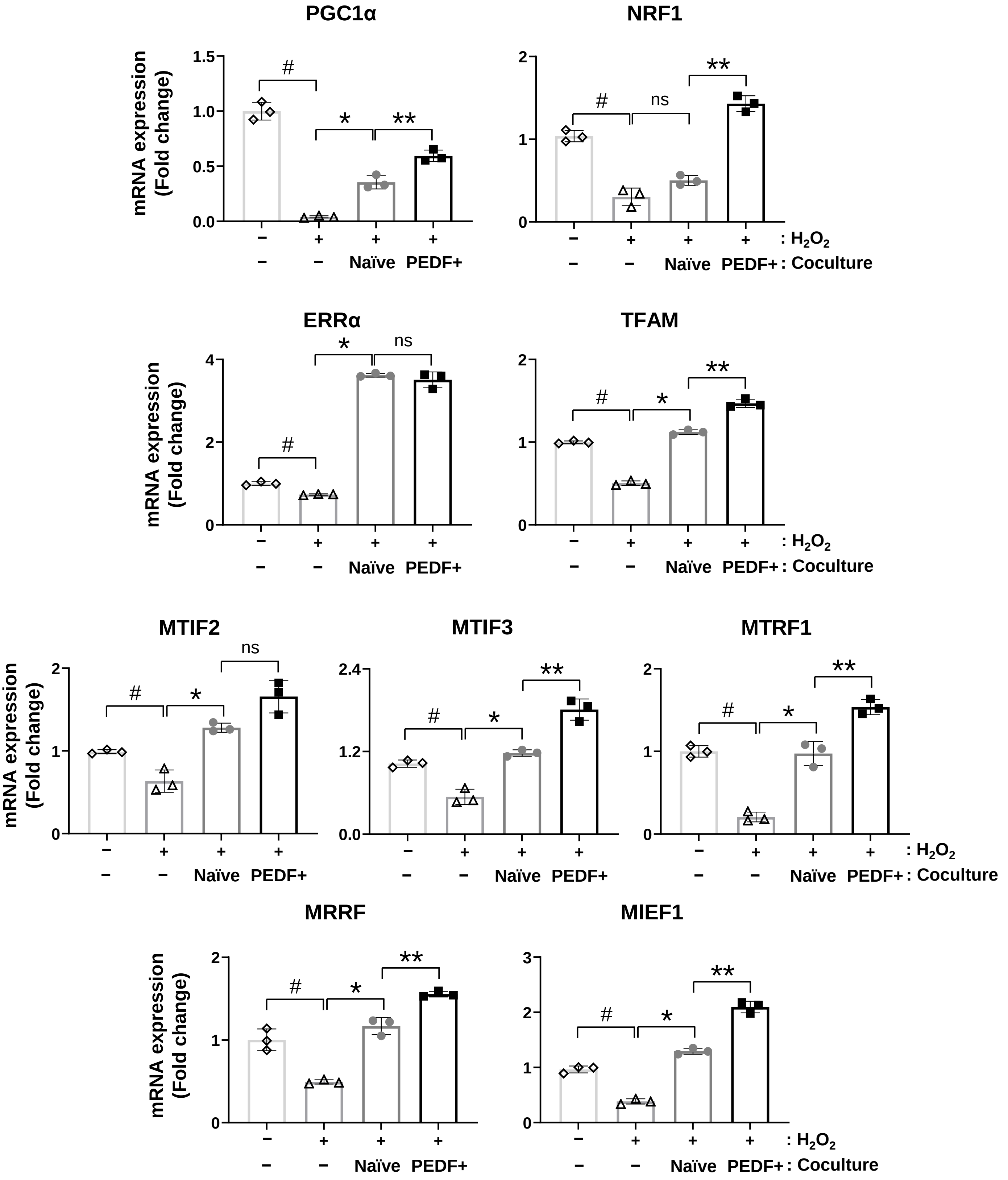

Figure S4. The mRNA expression of mitochondrial biogenesis (e.g., PGC1α, NRF1, ERRα, and TFAM) and translation (e.g., MTIF2, MTIF3, MTRF1, MRRF, and MIEF1) markers in ARPE-19 cells cocultured with naïve PD-MSCs (Naïve) or PD-MSCs^{PEDF} (PEDF+) for 24 hours by qRT-PCR. The data from each group are shown as the mean ± SD and were analyzed by Student's t-test or one-way ANOVA. #$p<0.05$ vs. the normal control (−), *$p<0.05$ vs. H_2O_2 treatment, **$p<0.05$ vs. naïve PD-MSCs.

Table S1

Gene	Accession number	Sequence (5' – 3')	Tm (℃)
PEDF	NM_001329903.2	F: 5'- CCCATGATGTCGGACCCTAA -3'<br>R: 5'- TGTCATGAATGAACTCGGAGGTG -3'	55
SIRT-1	NM_012238.5	F: 5'- GCCTCACATGCAAGCTCTAGTGAC -3'<br>R: 5'- TTCGAGGATCTGTGCCAATCATAA -3'	60
PGC1α	NM_013261.4	F: 5'- CAGCAAAAGCCACAAAGAC -3'<br>R: 5'- GGGTCAGAGGAAGAGATAAAGTTG -3'	60
NRF1	NM_005011.4	F: 5'- GCTTCAGAATTGCCAACCA -3'<br>R: 5'- GTCATCTCACCTCCCTGTAAC -3'	60
ERRα	NM_004451.5	F: 5'- GGCCCTTGCCAATTCAGA -3'<br>R: 5'- GGCCTCGTGCAGAGCTTCT -3'	60
TFAM	NM_003201.2	F: 5'- GAACAACTACCCATATTTAAAGCTCA -3'<br>R: 5'- GAATCAGGAAGTTCCCTCCA -3'	60
MTIF2	NM_001005369.1	F: 5'- GCCAAAGATGCACAGGTTCC -3'<br>R: 5'- AGCCATCAGATTATCGCCCG -3'	55
MTIF3	NM_001166263.2	F: 5'- GCACCAGCACAGTTGTCC -3'<br>R: 5'- CCCAAATCATTGCCCTTCTC -3'	55
MTRF1	NM_001354073.1	F: 5'- GATCCCTGAGGGGCTGCT -3'<br>R: 5'- TGCCTCAATTTTTCTTCCTCGG -3'	55
MRRF	NM_138777.5	F: 5'- GGGCGATAGCTGTGGATGTT -3'<br>R: 5'- CCTTTCCCTTTGGCTTTGGC -3'	55
MIEF1	NM_019008.6	F: 5'- TCGACACAGATACATTCTGCCC -3'<br>R: 5'- TCACCACCTGCAGGTCATCG -3'	55
RDH5	NM_001199771.3	F: 5'- CTGTGACCAACCTGGAGAGTCT -3'<br>R: 5'- GATGCGCTGTTGCATTTTCAGGT -3'	60
RDH11	NM_016026.4	F: 5'- AGCAGGTGTTGGTGCGGAAACT -3'<br>R: 5'- CGGACACATCATCACTCCTGCA -3'	60
LRAT	NM_004744.5	F: 5'- TGCGAGCACTTCGTGACCTACT -3'<br>R: 5'- GCCAATCCCAAGACTGCTGAAG -3'	60
RBP1	NM_002899.5	F: 5'- ACGGAGACAAGCTCCAGTGTGT -3'<br>R: 5'- GACCACACCTTCCACTCTCATC -3'	60
PINK1	NM_032409.3	F: 5'- GGACACGAGACGCTTGCA -3'<br>R: 5'- TTACCAATGGACTGCCCTATCA -3'	55
PARKIN	NM_004562.3	F: 5'- TGGAGGATTTAACCCAGGAG -3'<br>R: 5'- ACAGGGCTTGGTGGTTTTCT -3'	55
LC3	NM_022818.5	F: 5'- GAGAAGCAGCTTCCTGTTCTGG -3'<br>R: 5'- GTGTCCGTTCACCAACAGGAAG -3'	60
ATG7	NM_006395.3	F: 5'- CGTTGCCCACAGCATCATCTTC -3'<br>R: 5'- CACTGAGGTTCACCATCCTTGG -3'	60
GAPDH	NM_002046.7	F: 5'- GCACCGTCAAGGCTGAGAAC -3'<br>R: 5'- GTGGTGAAGACGCCAGTGGA -3'	60

Table S1. Human primer sequences using qRT-PCR.

Table S2

Gene	Accession number	Sequence (5' – 3')	Tm (℃)
PEDF	NM_177927	F: 5'- GATTGCCCAGCTGCCTTTGACA -3'<br>R: 5'-GGGACAGTCAGCACAGCTTGGATAG -3'	60
VEGF	NM_031836.3	F: 5'- ACGGACAGACAGACAGACAC -3'<br>R: 5'- CTTCTGGGCTCTTCTCTCTC -3'	55
RDH5	XM_008765056.3	F: 5'- TGGAGCCTGGCTTCTTTC -3'<br>R: 5'- GTAGTGGGCCTGTATAGCTG -3'	55
RDH11	NM_001012193.1	F: 5'- CCACAGCAAACTAGCCAACA -3'<br>R: 5'- CCAACTGGCAATCACTGAAA -3'	55
RDH13	NM_001108468.1	F: 5'- GCTGCCATGACCCTCATCAT -3'<br>R: 5'- CGTGGTCCAAACCAAAGCAG -3'	55
RDH14	NM_001109276.1	F: 5'- AAGAACTGCTACAGGAGGAGC -3'<br>R: 5'- CCCAGGTGGTTCACTCCAAA -3'	55
LC3	NM_022867.2	F: 5'- CGTCCTGGACAAGACCAAGT -3'<br>R: 5'- AGTGCTGTCCCGAATGTCTC -3'	55
ATG7	NM_001012097.1	F: 5'- GAGGAGACCGTCTGAGCAAC -3'<br>R: 5'- TGACACAGGAAAGGGTGCAA -3'	55
BECN1	NM_053739.2	F: 5'- TTTTCTGGACTGTGTGCAGC -3'<br>R: 5'- GCTTTTGTCCACTGCTCCTC -3'	55
MFN2	NM_130894.4	F: 5'- TTGGATGGACTATGCTAGTG -3'<br>R: 5'- TCCTCCGACCACGAGAATG -3'	52
PINK1	NM_001106694.1	F: 5'- CATGGCTTTGGATGGAGAGT -3'<br>R: 5'- TGGGAGTTTGCTCTTCAAGG -3'	55
PARKIN	NM_020093.1	F: 5'- CTGGCAGTCATTCTGGACAC -3'<br>R: 5'- CTCTCCACTCATCCGGTTTG -3'	55
GAPDH	NM_017008.4	F: 5'- TCCCTCAAGATTGTCAGCAA -3'<br>R: 5'- AGATCCACAACGGATACATT -3'	58

Table S2. Rat primer sequences using qRT-PCR.

Table S3

Antibody	Company	Catalog number
VEGF	Novus Biologicals	NB100-664
PEDF	Lsbio	LS-C818719
LC3	Cell signaling	NB100-2220
PINK1	Abcam	Ab23707
RDH11	Bioss	BS-6214R
ATG7	Santa Cruz Biotechnology	SC-8868
GAPDH	AbFrontier	LF-PA0018
Alexa Fluor 594 goat anti-rabbit IgG	Invitrogen	A11012
Anti-HRP-conjugated mouse IgG	Bio-rad	1705047
Anti-HRP-conjugated Rabbit IgG	Bio-rad	1721019

Table S3. Antibody list.
